# Risk factors for morbidity and death in non-cystic fibrosis bronchiectasis: a retrospective cross-sectional analysis of CT diagnosed bronchiectatic patients

**DOI:** 10.1186/1465-9921-13-21

**Published:** 2012-03-16

**Authors:** Pieter Christian Goeminne, Hans Scheers, Ann Decraene, Sven Seys, Lieven Joseph Dupont

**Affiliations:** 1 Laboratory of Pneumology, Katholieke Universiteit Leuven, Leuven, Belgium; 2 Department of Respiratory Medicine, University Hospital Gasthuisberg, Leuven, Belgium; 3 Department of Respiratory Medicine, University Hospital Gasthuisberg, Herestraat 49, 3000 Leuven, Belgium

**Keywords:** Bronchiectasis, Non-cystic fibrosis, Mortality, Morbidity, Risk factor

## Abstract

**Introduction:**

There is a relative lack of information about the death rate and morbidity of non-cystic fibrosis bronchiectasis and most studies are limited due to referral bias. We wanted to assess death rate and morbidity in those patients at our hospital.

**Methods:**

Adult patients seen at our department between June 2006 and November 2009 were recruited if the key string *"bronchiect*-" was mentioned in electronic clinical records and if chest CT imaging was available. Clinical records of all patients with confirmed radiologic diagnosis of bronchiectasis were reviewed and clinical characteristics were analyzed.

**Results:**

539 patients with a radiographic diagnosis of non-cystic fibrosis bronchiectasis were identified in a retrospective cross-sectional analysis giving a prevalence of 2.6% in our hospital population. A wide range of etiologies was found with idiopathic bronchiectasis in 26%. In the 41 months interval, 57 patients (10.6%) died. We found a median exacerbation rate of 1.94 per year. Bacterial colonization status was associated with more deaths, exacerbation rate, symptoms and reduced pulmonary function. Pulmonary hypertension was found in 48% of our patients.

**Conclusions:**

We evaluated a large non-cystic fibrosis bronchiectasis population, and provided new epidemiological data on associations between clinical characteristics and deaths and morbidity in these patients.

## Introduction

First described by Rene Theophile Laënnec in 1819, bronchiectasis (BX) are now defined as permanently dilated airways due to chronic bronchial inflammation caused by inappropriate clearance of various microorganisms and recurrent or chronic infection [1,2]. Diagnosing BX has become significantly easier with the advent of high resolution computed tomography (HRCT), which has proved to be highly sensitive for demonstrating bronchiectatic change in the airways [3]. Overall, postinfectious and idiopathic BX are the most frequent cause of non-CF bronchiectasis (NCFB), although the list of potential etiologies is extensive [4-6].

In the past, several studies evaluated clinical and microbiological characteristics of this NCFB population [5-13]. Although these studies identified a number of risk factors associated with lung function decline, the populations studied are limited due to referral bias. Most descriptive studies recruited patients with NCFB who were referred to their institution with a suspected or established clinical diagnosis of NCFB. As a result, the populations studied consist of a large number of patients with rare diseases and exclude certain subgroups such as smokers and patients with COPD [9-11]. Due to the heterogeneity of these study populations, the true burden of NCFB may be underestimated. There is a relative lack of information available about the mortality and morbidity of this condition. Such information is important as it allows us to prioritize treatment options and gain insights for proper design of clinical trials in NCFB. Our aim was to identify all patients with a radiologic diagnosis of BX in an unselected manner in order to assess mortality and morbidity in those patients with NCFB and to identify risk factors for mortality and morbidity.

## Methods

### Study population and patient assessment

We performed a search in the electronic patient file database of the University Hospital of Leuven, Belgium. This database lists patient records of all the patients seen at the University Hospital Leuven, which serves both as a local hospital and training center for the region of Leuven/Vlaams-Brabant as well as a national secondary and tertiary referral center. The search was done using these criteria: adult patients with the key string "*bronchiect*-" mentioned in the clinical records, with a contact at the department of Respiratory Medicine of our hospital between June the 1^st ^2006 (the furthest date we could go back in the electronic database) & November the 1^st ^2009 and who had CT imaging of the chest available. Approval was obtained from the local ethical committee of UZ Leuven, Belgium.

The chest CT scan images of all these patients were re-assessed to confirm/exclude the presence of BX. CT scans were performed by one of the UZ Leuven respiratory radiologists or at other centers. Images obtained using 1 mm collimation at full inspiration were reviewed and BX was deemed to be present if there was one or more of the following criteria: a bronchoarterial ratio greater than 1, lack of tapering of the bronchi and visualization of bronchi within 1 cm of costal or paravertebral pleura or abutting the mediastinal pleura [14].

In the patients with BX confirmed on chest CT scan, clinical records were retrospectively reviewed and the following characteristics were noted: age, sex, weight, age at diagnosis, smoking history, BX diagnostic investigations that were performed and/or final diagnosis listed, current respiratory symptoms at diagnosis and outside an exacerbation (presence/absence of chronic cough, sternal pain, wheezing, hemoptysis or shortness of breath; total number of respiratory symptoms (1 to 5)), the number of exacerbations the first year after CT based diagnosis of BX (defined as the need for antibiotic treatment due to increased respiratory symptoms), presence of gastro-esophageal reflux disease (GERD)(defined as presence of esophagitis on gastroscopy and/or an abnormal 24 hour pH monitoring), presence of sinusitis (defined as an abnormal sinus CT scan and/or suggestive symptoms such as postnasal drip, chronic (purulent) discharge from the nose or symptoms of pain or tenderness over one of the sinuses) and the presence of pulmonary hypertension (PH)(defined as a systolic pulmonary arterial pressure of ≥36 mmHg on echocardiography assuming normal right atrial pressure of 5 mmHg) [15]. All sputum culture results obtained during the study period were reviewed and bacteria found since the diagnosis of BX were registered even during an exacerbation. All patients underwent spirometry according to ATS criteria [16-18] and the best results at diagnosis and outside an exacerbation were noted. Follow-up was by the different outpatient pulmonary clinics and depended on severity and stage of the underlying etiology and disease with a minimum of once per year. Standard follow-up at the NCFB outpatient clinic is once every three to four months.

### Statistics

Associations between characteristics were analyzed using a broad set of statistical tests. Logistic, Poisson and linear regression models were used for binary, count and continuous outcomes respectively. Chi square tests with calculation of odds ratios (OR) were used for frequency distributions and non-parametric tests such as Mann-Whitney U test were chosen when the assumption of normal distribution was violated. Data were analyzed with GraphPad Prism4.01 and SAS9.1.3. The p-values reached significance if lower than 0.05 and two-tailed testing was performed.

## Results

### Patient characteristics

**Figure 1:**
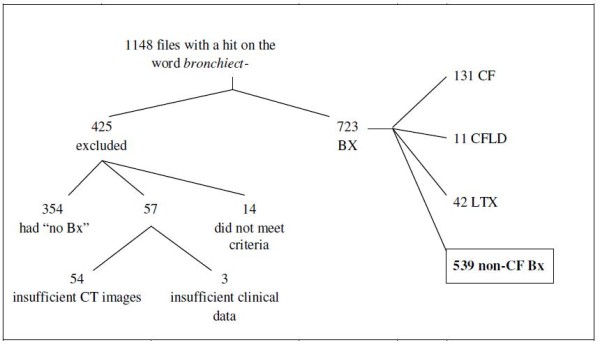
**Patient selection algorithm of adult patient files reviewed**. CF = Cystic fibrosis; CFLD = CF Like Disease; BX = Bronchiectasis; CT = Computer Tomography; LTX = Lung transplant patients; non-CF Bx = Non-Cystic Fibrosis Bronchiectasis.

The patient selection algorithm is shown in Figure 1 and Additional file 1: Supplement text S1. Further analysis and chart review was done in this population of 539 NCFB patients. Patient characteristics are listed in Table 1.

**Table 1:** Demography and smoking status of the studied population

Male/Female ratio	299/240 (55.5%/44.5%)
Age (years)	median 67; p_25-75 _57-75
Age at diagnosis (years)	median 62; p_25-75 _51-71
BMI (kg/m²) at diagnosis	median 24; p_25-75 _21-27
Active smoker	69/539 (12.8%)
Ex-smoker	185/539 (34.3%)
Never smoked	268/539 (49.7%)
Passive smoker only	17/539 (3.1%)
FVC (% pred)	mean 83% ± 23%
FEV_1 _(% pred)	mean 67% ± 25%
TLC (% pred)	mean 92% ± 18%
RV (% pred)	mean 116% ± 42%
TLCO (% pred)	mean 62% ± 21%
Exacerbations/year	1.94 (median 1; p_25-75 _1-3; range 0-12)

Middle column shows absolute numbers and percentages are listed in the brackets. *BMI *= Body Mass Index; *FEV_1 _*= Forced expiratory volume in one second; *FVC *= Forced vital capacity; *RV *= Residual Volume; *p *= percentile; *pred *= predicted; *TLC *= Total Lung Capacity; *TLCO *= Transfer Factor of the Lung for Carbon Monoxide

**Figure 2:**
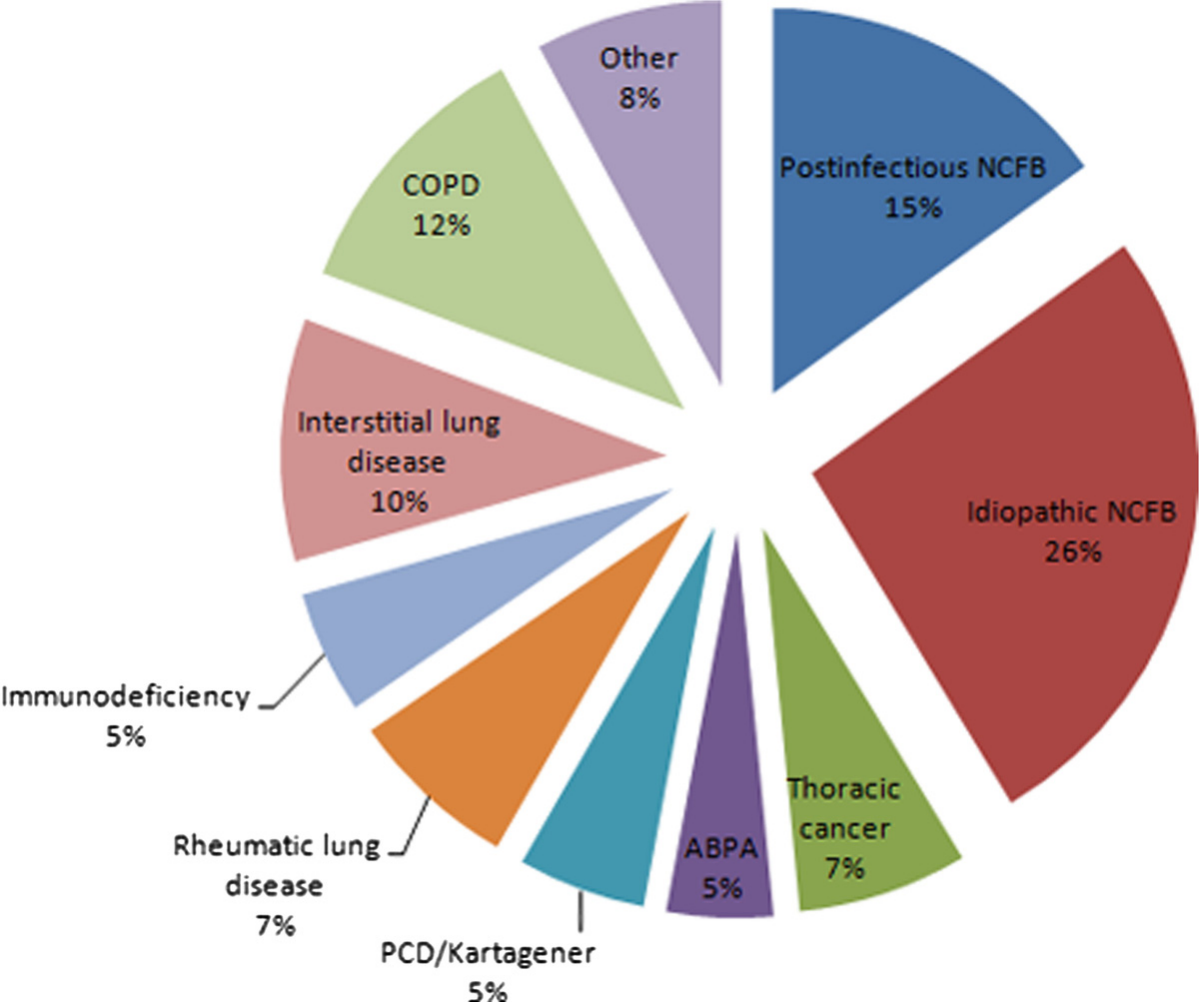
**Underlying etiology of the studied population**. ABPA = Allergic Bronchopulmonary Aspergillosis; COPD = Chronic Obstructive Lung Disease; PCD = Primary Ciliary Dyskinesia, NCFB = Non Cystic Fibrosis bronchiectasis

Patient medical files were reviewed to identify the underlying etiology. We did not find a clear underlying cause for the BX in 170 patients. In 22 of these patients there were two possible diagnoses and for the subgroup analysis, patient data were included in both diagnostic subgroups. 148 patients lacking a final diagnosis, although full diagnostic work-up had been performed, were labeled as having idiopathic NCFB. The underlying etiology of the BX is shown in Figure 2 and Table 2. Analysis showed that BX due to thoracic cancer (OR = 3; p = 0.004), COPD (OR = 4; p < 0.0001) and interstitial lung disease (OR = 2; p = 0.02) were mostly male. Patients with idiopathic BX were predominantly female (OR = 2; p < 0.0001).

**Table 2:** Further definition of the underlying NCFB etiologies

Postinfectious (84)	TB (37), MAC (5), Pertussis (3), Invasive Aspergillosis (2), Measles (1), Diphtheria (1), *Blastocystes hominis *(1), unkown (34)
COPD (64)	GOLD II (10), GOLD III (28), GOLD IV (26)

Interstitial lung disease (57)	EAA (15), IPF/NSIP (27), BOOP (9), drug induced ILD (3), UIP (3)

Rheumatic lung disease RA (29), Sjögren (5), Sclerodermia (4), AS (1), MCTD (1) (40)	

Immunodeficiency (29)	CVID (1), HIV (1), hyper IgE syndrome (1), other immunoglobulin deficits (14 dysgammaglobulinemia, 1 agammaglobulinemia, 2 hypogamma-globulinemia), Navajo poikiloderma (1), different types of post transplants (5) (3 kidney, 1 liver and 1 heart), non-thoracic Non-Hodgkin lymphoma (2), chronic lymphatic leukemia (1)

Thoracic cancer (38)	adenocarcinoma (9), large cell anaplastic carcinoma (1), squamous cell carcinoma (16), small-cell lung carcinoma (3), thoracic Non-Hodgkin lymphoma (3), carcinoid (1), mesothelioma (2), solitary pulmonary nodule without tissue diagnosis (3)

Other causes (44)	sarcoidosis (13), anatomic malformations (6), UC (6), Crohn (3), SJM (3), aspiration/inhalation (3), α_1_-ATD (2), CS vasculitis (2), Wegener (2), MK (1), PE (1), WC (1), Microscopic polyangiitis (1)

The number in brackets states the number of patients. *AS *= Ankylosing Spondylitis, *α_1_*-*ATD *= alfa-1-antitrypsin deficiency, *BOOP *= Bronchiolitis Obliterans Organizing Pneumonia, *COPD *= Chronic Obstructive Lung Disease, *CS *= Churg-Strauss, *CVID *= Common Variable Immunodeficiency, *EAA *= Extrinsic Allergic Alveolitis, *ILD *= Interstitial Lung Disease, *IPF *= Idiopathic Pulmonary Fibrosis, *MAC *= Mycobacterium avium complex, *MCTD *= Mixed Connective Tissue Disease, *MK *= Mounier-Kuhn Syndrome, *NSIP *= Nonspecific Interstitial Pneumonia, *PE *= Pseudoxanthoma Elasticum, *RA *= Rheumatoid Arthritis, *SJM *= Swyer-James-McLeod Syndrome, *TB *= Tuberculosis, *UC *= Ulcerative Colitis, *UIP *= Usual Interstitial Pneumonia, *WC *= Williams-Campbell syndrome

During the study period, a total of 20,998 patients were seen at least once in the department of adult Respiratory Medicine of which 539 had a radiological diagnosis of NCFB, giving an estimated prevalence of 2.6% amongst the overall patient population seen for respiratory problems at our hospital.

### Symptoms

There was more sinusitis in NCFB patients with Primary Ciliary Dyskinesia (PCD) and idiopathic BX and less in patients with COPD, thoracic cancer and interstitial lung disease. Patients with sinusitis had more different species of bacteria present in sputum (p < 0.0001) with significance for *PA *(P = 0.0026; OR = 1.88), encapsulated *Pseudomonas aeruginosa (PA) *(p = 0.0013; OR = 2.35) and *Staphylococcus aureus (SA) *(p = 0.0063; OR = 1.85). Patients with sinusitis had more symptoms than others (p = 0.0004) with a significant association between sinusitis and cough (p = 0.01; OR = 1.93), wheezing (p = 0.033; OR = 1.57) and sternal pain (p = 0.0247; OR = 1.65), independent of the underlying etiology. Other results on symptoms can be seen in Additional file 1: Supplement text S2.

### Gastro-esophageal reflux

Signs of GERD were present in 28% of our patients with NCFB. There was no significant difference in the presence of GERD between patients with different NCFB etiologies. The presence of shortness of breath was significantly associated with GERD (p = 0.001; OR = 2.4) and remained significant after correcting for underlying etiology. Cough, when controlled for etiology was also significantly associated with GERD (p = 0.0162; OR = 1.98). Overall, the number of symptoms in patients with GERD was higher than in patients without documented GERD (p = 0.0051). These patients with GERD also had more different bacteria present in their sputum cultures than patients without GERD (p = 0.016). The presence of *SA *(p = 0.0007; OR = 2.16) but not *PA *(p = 0.068; OR = 1.48) was associated with the presence of GERD.

### Bacterial colonization

**Figure 3:**
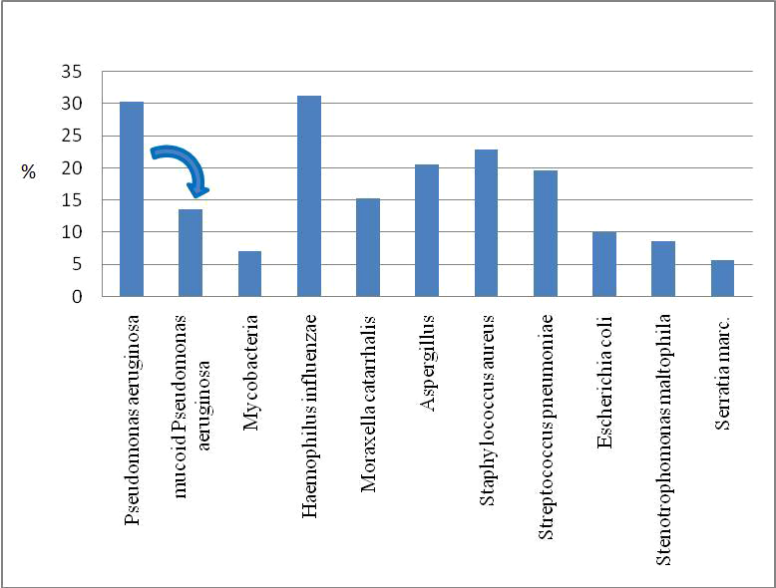
**Percentage of bacteria present in sputum or bronchoalveolar lavage culture of NCFB patients**. Only bacteria with a prevalence of more than 5% are shown

Sixty patients did not have any record of sputum culture or bronchoalveolar lavage (BAL) culture during the study period. The sputum culture results of the other patients are summarized in Figure 3. The main bacteria found are *PA *in 30%, *Haemophilus influenzae *in 31%, *SA *in 23%, *Aspergillus fumigatus *in 20%, *Streptococcus pneumonia *in 20% and *Moraxella catharralis *in 15% of the patients. Other bacteria found are listed in Additional file 1: Supplement text S3.

Underlying etiology was associated with the number of total different bacteria cultured in sputum. Idiopathic BX (p = 0.003), sarcoidosis (p = 0.03), rheumatic (p = 0.05) and interstitial lung disease with BX (p < 0.0001) had less often bacteria present in sputum while bacteria were found more often in sputum cultures of patients with ABPA (p = 0.001), PCD (p = 0.001) and COPD (p < 0.0001). Subanalysis showed that *PA, Moraxella cattharalis*, *Streptococcus pneumonia *and *Escherichia coli *were seen significantly more often in COPD patients and the former three significantly less in patients with interstitial lung disease (p = 0.001). As expected, *Aspergillus *was present more often in patients with ABPA (p = 0.0003).

### Lung function

The mean spirometry values at diagnosis are shown in Table 1. Spirometry results were related to the underlying etiology (FEV_1 _p < 0.0001; FVC p = 0.008) with a significantly lower FEV_1 _in COPD patients as compared to the NCFB patients with other etiologies (p < 0.0001). There was a significant negative association between FEV_1 _and number of respiratory symptoms, with a decrease of 104 ml (p = 0.0063) or 3.7% (p = 0.0012) per symptom. Airway resistance and residual volume were correlated with total number of bacteria in sputum, while FEV_1 _and FVC were negatively correlated with total number of cultured bacteria (all: p < 0.0001). A worse lung function was associated with presence of *PA *(p < 0.0005), *Haemophilus influenzae *(p = 0.05), *Streptococcus pneumonia *(p = 0.05), *SA *(p = 0.05), *Moraxella catharralis *(p = 0.001) and *Stenotrophomonas maltophilia *(p = 0.01).

### Pulmonary hypertension

Echocardiography data were available in 289 of the 539 patients (53.6%). In total 140 of the 289 (48.4%) patients were diagnosed with PH, with information about their systolic pulmonary arterial pressure in 130 patients (without the right atrial pressure of 5 mmHg: median 37.5 mmHg; p_25-75 _33-48). We found that 10% of the patients with PH died during the study interval of 41 months. There was no significant correlation between the presence of PH and the colonization status or the number of exacerbations. There was a difference in presence of PH between the different etiologies (p = 0.012): there were more NCFB patients with PH in the COPD group and less patients with PH in the postinfectious group. The severity of PH (systolic pulmonary artery pressure in mmHg) was not significantly different between different etiologies (p = 0.067). Patients with PH had worse FEV_1 _than patients without PH (p = 0.01), with a trend for FVC (p = 0.08). There was a significant inverse correlation between the systolic pulmonary artery pressure (mmHg) and FEV_1_, FVC, TLC, RV and TLCO (Additional file 1: Table S4). The significance remained after correcting for underlying etiology, with the exception of RV.

### Exacerbation rate

Yearly exacerbation rate is listed in Table 1. Factors associated with more exacerbations were sinusitis (p < 0.0001), GERD (p = 0.048), low FEV_1 _(p = 0.0003; r = -0.15) number of different bacteria cultured in sputum (p < 0.0001;r = 0.34), presence of (encapsulated) *PA *(both p < 0.0001) and *SA *(p = 0.0003) in sputum culture. Etiologies associated with an increased exacerbation rate were PCD (p < 0.0001), immunodeficiency (p < 0.0001), anatomical malformations (p < 0.0001) and COPD (p = 0.049). Fewer exacerbations were noted in patients with thoracic cancer (p = 0.015), interstitial lung disease (p < 0.0001) and sarcoidosis (p = 0.0003).

### Death rate

During the 41-month period, 57 patients died (10.6%) (Additional file 1: Figure S5). Subgroup analysis showed lower 41 month deaths in patients with idiopathic or postinfectious BX (3.4% and 7.1% respectively) with significance for idiopathic NCFB (OR = 0.23; p = 0.0008) as opposed to other etiologies. Patients with thoracic cancer and COPD had the highest deaths (30% and 26% respectively; OR = 4.32 and 3.93; both p < 0.0001). More deaths were also seen in active smokers (OR = 1.98; p = 0.05) and in former smokers (OR = 1.87; p = 0.045). Death was associated with number of different bacteria in sputum cultures, with more deaths when more different species were present (p = 0.002). Only *Escherichia coli *(p = 0.0003) and *Aspergillus species *(p = 0.034) showed significant association with death but trends were seen for *PA* (p = 0.077), *Haemophilus influenzae *(p = 0.11), *SA* (p = 0.11) and *Moraxella catharralis *(p = 0.056). The number of symptoms was not associated with death and neither was the number of exacerbations. Low FEV_1 _and FVC (p = 0.0008 & p = 0.002, respectively) and presence of PH (p = 0.01) were associated with more deaths.

## Discussion

Radiological diagnosis NCFB was confirmed in a total of 539 patient which results in a NCFB prevalence of 2.6% in the patient population of the department of respiratory medicine at the University Hospital of Leuven, Belgium. The use of the electronic medical records database of our hospital to capture potential patients and thorough review of CT scan and clinical data allowed us to identify NCFB patients in an unselected manner. We are confident that we have obtained a nearly full estimate of patients with NCFB at our hospital, which explains the larger number of patients than similar studies reported in the literature. The setting of our study does not allow us to give an exact population estimate of NCFB, which has been estimated to be much lower than our data, ranging from 52 per 100 000 in the overall US population to 1 per 625 Auckland Pacific Island children [19-21]. Even incidence estimates fall within this range, running from 3.7 to 17.2 per 100 000 per year [22-24].

Our database included almost all possible underlying etiologies in BX [4]. Our population included similar numbers of idiopathic BX as reported in other cohort studies but for other etiologies, the number of patients differed somewhat [5-13]. This discrepancy may be due to the fact that patients with NCFB were recruited on the basis of radiological evidence of BX and not only on the basis that they were referred to our hospital because of NCFB. As a result, patients with NCFB due to COPD, ILD or thoracic cancer were also recruited. Gender distribution in our study population showed a small male predominance, which is due to the inclusion of patients with NCFB due to COPD, thoracic cancer and ILD. Previous studies suggested that chronic airway infection due to NCFB was a more common and more virulent disease in women [5-11,13,25]. In our study population, patients with idiopathic BX were predominantly female.

Our 41 month death rate of 10.6% is in line with the findings of Loebinger et al. who found survival rates of 91% at 4 years [26]. We found a lower death rate in idiopathic BX, indicating that a lack of underlying etiology of NCFB bears a favorable prognosis. One possible explanation for this marked difference could be the absence of comorbidities in patients with idiopathic NCFB as compared to etiologies such as COPD and rheumatic disease. Another possible explanation might be that patients with idiopathic BX have a less severe pathophysiological vicious cycle of impaired mucociliary clearance, infection and inflammation and as a result develop lung damage more gradually than other known etiologies. In a study by Reiff et al., patients with idiopathic BX were shown to have less extensive disease on CT images than other etiologies, which is in agreement with our findings [27]. King et al. found that patients with idiopathic BX had a low neutrophil oxidative burst, which may result in less severe airway inflammation [28]. Children with idiopathic BX had less viscous sputum than children with CF or chronic bronchitis, which may lead to improved cough transportability of sputum [29]. Additional research is needed to unravel the pathogenesis of idiopathic BX. Our finding of a high presence of sinusitis in this subgroup suggests an upper airways dysfunction.

In our study population, we observed an exacerbation rate of nearly 2 exacerbations per year, indicative of a significant burden of disease in patients with NCFB. This exacerbation rate was slightly higher than previously reported by O'Donnell et al. (1.5 exacerbations per year in patients with idiopathic BX) [30]. The higher exacerbation rate is probably due to the inclusion of other etiological groups such as COPD, where recent research has shown a difference in oxidative stress in the lungs of COPD patients versus BX patients. Tzortzaki et al. showed a higher specific sputum marker of oxidant-induced DNA damage in COPD patients versus BX patients [31]. Another reason for differences in reported exacerbation rate might be the criteria we used to define exacerbation, given the lack of a clear definition of a NCFB exacerbation. In the current study, an exacerbation was defined not merely on the basis of symptoms, clinical or radiologic changes but on the need for a therapeutic intervention with antibiotics (oral or IV). Interstitial lung disease, sarcoidosis and thoracic cancer showed a lower exacerbation rate. These patients also had a lower number of symptoms and lower number of different bacteria in sputa, explaining their lower exacerbation rate. They could be regarded as having 'dry' bronchiectasis, a term previously used to describe patients with BX but without expectoration of a lot of sputa [32].

Our data showed a significant negative correlation between FEV_1 _and exacerbation rate and the FEV_1 _was on an average 104 ml or 3.7% lower per symptom reported by our patients. This is in line with Wilson et al. who found that lung function correlated with St.-George's Respiratory Questionnaire (SGRQ) [33].

In our population of NCFB patients 48% of the patients with an echocardiographic evaluation had evidence of PH and the presence of PH was associated with more severe lung disease (decrease in lung function parameters). These data are in agreement with similar study data that showed that PH was present in one out of three patients with NCFB and correlated with radiographic stage [34]. Alzeer et al. saw that right ventricular dysfunction and right ventricular dimensions were greater in cystic BX and were positively correlated with systolic pulmonary artery pressure and negatively correlated with partial pressure of oxygen in arterial blood. In more severe BX disease, there is an impaired perfusion with more capillary bed destruction and left-to-right shunt, leading to impaired cardiac function and pulmonary gas exchange [34]. This might explain the bad prognostic factor of PH in NCFB. Due to the high prevalence of PH, cardiac assessment with echocardiography is indicated in patients with NCFB and more severe lung disease. There was no association between PH and other clinical characteristics. Despite these findings, PH in BX did not have a significant impact on death in our population.

The main advantage of our study is the large patient sample and the unbiased recruitment with as main inclusion criterion the radiological documentation of BX. Not only those patients who were referred to our center for management of their NCFB were recruited but also patients with other underlying diseases, in whom the presence of BX was perhaps considered to be a confounding or complicating factor. The main limitation of this study is its retrospective design. All clinical data were collected by reviewing patient medical records, leaving error for underreporting. Despite these disadvantages, data are in line with previous studies. Previous reports suggest that a triad of mucus plugging in the airways, infection and inflammation may reinforce each other, lead to progressive lung damage and result in mortality and morbidity [23]. It is clear that BX remains a significant health problem, requiring further research [35]. Our study revealed some additional factors influencing death and morbidity and may help to further clarify the different elements that are important in NCFB (Additional file 1: Table S6 [36]). The association between the bacterial colonization status and several clinical outcome parameters suggest the potential importance of anti-infective treatment. Dhar et al. reported their experiences with nebulised colistin in a small cohort of patients with NCFB infected with *PA*. Regular colistin reduced the exacerbation and hospitalization rate, a finding which needs confirmation in larger controlled trials [37].

## Conclusion

In summary, NCFB was confirmed radiologically in nearly 3% of the patients seen at the department of respiratory medicine of our hospital with a wide spectrum of underlying etiologies. Although the general death rate of our NCFB population is comparable to literature data, we found that idiopathic BX showed lower death rate, emphasizing that a full work-up for NCFB may have prognostic implications. Repetitive bacterial follow-up with sputum culture is necessary as presence of *PA *was high and culture results were correlated with other factors such as symptoms, lung function, exacerbation rate and death rate. Presence of sinusitis and GERD was also associated with an increased exacerbation rate. Our analysis showed PH in 48% of the patients, underlining the need for echocardiographic evaluation in patients with BX.

## Abbreviations

ABPA: Allergic broncho-pulmonary aspergillosis; ANOVA: Analysis of variance; BX: Bronchiectasis; CF: Cystic fibrosis; CFLD: Cystic fibrosis like disease; COPD: Chronic obstructive pulmonary disease; CT: Computed tomography; FEV_1_: Forced expiratory volume in one second; FVC: Forced vital capacity; GERD: Gastro-esophageal reflux; HRCT: High resolution computed tomography; IBD: Inflammatory bowel disease; ILD: Interstitial lung disease; IV: Intravenous; NCFB: Non-cystic fibrosis bronchiectasis; OR: Odds ratio; PA: *Pseudomonas aeruginosa*; P_25-75_: Percentile 25 and 75; PCD: Primary Ciliary Dyskinesia; PH: Pulmonary hypertension; RA: Rheumatoid arthritis; RV: Residual volume; SA: *Staphylococcus aureus; *SGRQ: St.-George's respiratory questionnaire; TLC: Total lung capacity; TLCO: Transfer factor of the lung for carbon monoxide.

## Conflict of interests

None of the authors have a financial relationship with a commercial entity that has an interest in the subject of the presented manuscript.

## Authors' contributions

PG performed the acquisition and analysis of the data, designed the study and wrote the manuscript. HS processed the data and performed part of the analysis. AD and SS were involved in the critical revision of the manuscript prior to submission. LD was involved in the design and critical revision prior to submission. All authors read and approved the final manuscript.

## Supplementary Material

Additional file 1 **Supplementary data text S1 shows patient selection algorithm; text S2 describes extra data on symptoms; text S3 gives additional information on bacteria found; table S4 shows correlation between lung function and severity of pulmonary hypertension; figure S5 shows deaths in non-cystic fibrosis bronchiectasis; table S6 indicates the associations between the different morbidity factors**. Click here for file
