# Supplementary material for: Risk factors for morbidity and death in non-cystic fibrosis bronchiectasis: a retrospective cross-sectional analysis of CT diagnosed bronchiectatic patients
Source: Respir Res. 2012 Mar 16;13(1):21. doi: 10.1186/1465-9921-13-21 (PMC3379934; doi:10.1186/1465-9921-13-21)
Supplement: Additional file 1 — Supplementary data text S1 shows patient selection algorithm; text S2 describes extra data on symptoms; text S3 gives additional information on bacteria found; table S4 shows correlation between lung function and severity of pulmonary hypertension; figure S5 shows deaths in non-cystic fibrosis bronchiectasis; table S6 indicates the associations between the different morbidity factors. [file 1465-9921-13-21-S1.DOC]

**Risk factors for morbidity and death in non-cystic fibrosis bronchiectasis: a retrospective cross-sectional analysis of CT diagnosed bronchiectatic patients**

Pieter Christian Goeminne (MD), Hans Scheers (MSc), Ann Decraene (MSc), Sven Seys (MSc), Lieven Joseph Dupont (MD, PhD)

**Online Data Supplement**

**Supplement text S1**

*Patient selection algorithm*

In total, 1148 patient files had the word *bronchiect-*. Of these 1148 patients, 354 had the statement of “*no bronchiectasis present*” in their records and were subsequently excluded. Of the remaining 794 patients, 54 did not have sufficient CT images available to assess the presence of bronchiectasis (BX), three had insufficient clinical data and 14 were labeled as having BX, but in fact did not meet the criteria for BX. One patient was included although he did not have CT but Magnetic Resonance Imaging, also effective for the diagnosis and scoring of BX [S1]. The remaining population comprised 131 patients with CF, 11 with CF Like Disease (CFLD), 42 patients who were transplanted (Ltx) for reasons other than CF or CFLD and a remainder of 539 patients with non-cystic fibrosis BX (NCFB) (Figure 1).

|  | 1148 files with a hit on the word *bronchiect-* | | |  |  | 131 CF  11 CFLD  42 LTX  **539 non-CF Bx** |
| --- | --- | --- | --- | --- | --- | --- |
| 425 excluded | | |  | 723 BX |  |
| 354  had “no Bx” | 57 | | 14  did not meet criteria |  |  |
| 54 insufficient CT images | | 3 insufficient clinical data | |  |  |

**Figure 1**

Patient selection algorithm of adult patient files reviewed. CF = Cystic fibrosis; CFLD = CF Like Disease; BX = Bronchiectasis; CT = Computer Tomography; LTX = Lung transplant patients.

**Supplement text S2**

Chronic cough was present in 79% of patients and periods of shortness of breath in 78% of NCFB patients. A smaller proportion of patients had symptoms of wheezing (24%), hemoptysis (16%) or sternal pain (20%). There was a significant difference in total sum of symptoms between different NCFB etiologies (ANOVA:p=0.025) with the highest number of symptoms in PCD and rheumatic causes and the lowest in lung cancer and immunodeficiency. The total number of bacteria in sputa was higher in patients with symptoms of hemoptysis (p=0.0006) than without hemoptysis and those patients also had more often *PA* (p=0.088;OR=1.52), encapsulated *PA* (p=0.0015;OR=2.51) and *SA* (p=0.008;OR=1.98) present in their sputum culture.

**Text** **S3**

*Other bacteria found*

Other bacteria (with the total number of patients between brackets) were *Providencia spp.* (5), other *Pseudomonas spp.* (9), *Klebsiella spp.* (37), other *Streptococcus spp.* (6), *Coagulase negative Staph.* (9), *Enterobacter spp.* (24), *Pasteurella multocida* (8), *Proteus spp.* (23), *Morganella morganii* (13), *Burkholderia cepacia* (1), *Citrobacter spp.* (10), *Penicillium* (12), *Serratia* *liquefaciens* (4), *Neisseria* (1), *Achromobacter* *xylosoxidans* (7), *Alcaligenes* *xylosoxidans* (5), *Comamonas* *acidovirans* (1), *Hafnia* *alvei* (3), *Nocardia* *nova* (1), *Corynebacterium* spp. (1) and *Elizabethkingia* *meningoseptica* (1).

**Table S4**

| **Correlation between lung function and severity of pulmonary hypertension** | | | |
| --- | --- | --- | --- |
| **Parameter** | **Association** | **Parameter** | **Association** |
| FEV_1_ (Liter) | p=0.010; r=-0.18 | FVC (Liter) | p=0.009; r=-0.19 |
| FEV_1_ (% pred.) | p=0.13; r=-0.11 | FVC (% pred) | p=0.06; r=-0.13 |
| TLC % (Liter) | p=0.002; r=-0.24 | TLCO (% pred) | p<0.0001; r=-0.36 |
| RV (% pred) | p=0.026; r=-0.18 | Raw (% pred) | p=0.35; r=0.08 |
| RV (Liter) | p=0.07; r=-0.15 | Tiff | p=0.72; r=-0.025 |

**Table S4**

Correlation between lung function parameters and PH (mmHg measured by echocardiography). Raw= airway resistance; RV= residual volume; Tiff= FEV_1_/FVC;TLC= total lung capacity; TLCO= diffusing capacity of the lungs for carbon monoxide

**Figure** **S5**

*Deaths in NCFB*


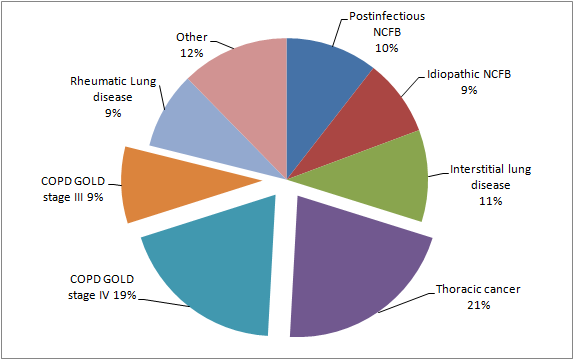


**Figure S5**

Underlying etiology of the NCFB patients that died within the 41-month study period. GOLD = Global Initiative for Chronic Obstructive Lung Disease, NCFB = Non Cystic Fibrosis bronchiectasis. Other = Allergic Bronchopulmonary Aspergillosis (1.7%), Primary Ciliary Dyskinesia (1.7%), Immunodeficiency (5.3%), Sarcoidosis (1.7%) and α_1_-antitrypsin deficiency (1.7%).

**Table S6**

|  | **Etiology**  **(as compared to the other etiologies)** | **Exacerbactions** | **FEV_1_** | **Symptoms** | **Sinusitis** | **Reflux** | **PH** | **Bacterial colonization** | | |
| --- | --- | --- | --- | --- | --- | --- | --- | --- | --- | --- |
|  |  |  |  |  |  |  |  | **# bacteria** | **PA** | **SA** |
| **Exacerbactions** | ✓ + : PCD, ID, AM, COPD  ✓ - : Tumor, ILD, SARC |  | ✓ - | ✓ + | ✓ + | ✓ + | 🗶 | ✓ + | ✓ + | ✓ + |
| **FEV_1_** | ✓ - : COPD |  |  | ✓ - | ✓ + | 🗶 | ✓ - | ✓ - | ✓ - | ✓ - |
| **Symptoms** | Highest #: PCD, rheumatic  Lowest #: Tumor, ID |  |  |  | ✓ + | ✓ + | 🗶 | ✓ + | ✓ + | ✓ + |
| **Sinusitis** | ✓ + : PCD, Idio  ✓ - : COPD, ILD, Tumor |  |  |  |  | ✓ + | 🗶 | ✓ + | ✓ + | ✓ + |
| **Reflux** | 🗶 |  |  |  |  |  | 🗶 | ✓ + | ✓ - | ✓ + |
| **PH** | ✓ + : COPD  ✓ - : PI |  |  |  |  |  |  | 🗶 | 🗶 | 🗶 |

**Table S6: Associations between different morbidity factors:** ✓ indicates an association and 🗶 indicates no association. + indicates a positive association and – indicates a negative association. AM = Anatomic Malformations; COPD = Chronic Obstructive Lung Disease; ID = Immunodeficiency; Idio = Idiopathic; ILD = Interstitial Lung Disease; PA = *Pseudomonas* aeruginosa; PCD = Primary Ciliary Dyskinesia; PH = pulmonary hypertension; PI = postinfectious; SA = *Staphylococcus aureus*; SARC = Sarcoidosis; # = number

Reference List

S 1 Montella S, Santamaria F, Salvatore M, Pignata C, Maglione M, Iacotucci P, Mollica C. Assessment of chest high-field magnetic resonance imaging in children and young adults with noncystic fibrosis chronic lung disease: comparison to high-resolution computed tomography and correlation with pulmonary function. *Invest Radiol* 2009;44:532-8.
